# A IgG4-related prostatitis case

**DOI:** 10.1093/omcr/omaf304

**Published:** 2026-02-18

**Authors:** Weizhe Han, Fang Yu, Nihati Rexiati, Jiantao Xiao, Zhonghua Yang

**Affiliations:** Department of Urology, Zhongnan Hospital of Wuhan University, Wuhan, P.C 430000, China; Department of Pathology, Zhongnan Hospital of Wuhan University, Wuhan, P.C. 430000, China; Department of Urology, Zhongnan Hospital of Wuhan University, Wuhan, P.C 430000, China; Department of Urology, Zhongnan Hospital of Wuhan University, Wuhan, P.C 430000, China; Department of Urology, Zhongnan Hospital of Wuhan University, Wuhan, P.C 430000, China

**Keywords:** immunology, sexual and reproductive health, radiology, nuclear medicine, and medical imaging, allergy

## Introduction

IgG4-related disease (IgG4-RD) is a well-established fibroinflammatory disease characterized by tumor-like lesions in affected organs, dense lymphoplasmacytic infiltrates rich in IgG4-positive plasma cells, storiform fibrosis, and elevated serum IgG4 concentrations [1].

Originally regarded as a digestive system-specific autoimmune disorder (primarily involving the pancreas), IgG4-RD was reclassified as a systemic disease in 2003 following the confirmation of extrapancreatic manifestations [2]. Prostatic involvement with associated symptomatology remains exceedingly rare in IgG4-RD. Given the absence of reliable biomarkers for IgG4-RD, histopathological examination remains the cornerstone of diagnosis.

## Case presentation

A 69-year-old Asian male with a history of grade 2 hypertension (no comorbidities such as diabetes) was incidentally diagnosed with thyroid nodules during a routine examination in late 2024, which remained untreated. He presented for further evaluation in March 2025. Ultrasound revealed bilateral thyroid colloid cysts, prompting hospitalization. Physical examination identified a 1 × 1 cm left cervical mass with firm consistency, non-tender, and no adhesion to surrounding tissues, without thyroid dysfunction or other discomforts. Concurrent urinary retention necessitated urological prioritization following multidisciplinary consultation; conservative management of thyroid pathology was initiated, with Foley catheter placement for acute urinary tract decompression.

Laboratory investigations during hospitalization showed:

- Total Prostate-specific antigen(PSA): 0.274 ng/ml (reference: < 4.0 ng/ml)
- Neuron-specific enolae (NSE): 12.1 ng/ml (reference: <  16.3 ng/ml)
- Carcino-embryonic antigen (CEA): 2.6 ng/ml (reference: < 7.2 ng/ml)
- Leukocytes: 13.45 × 10^9^/l
- eosinophils: 2.33 × 10^9^/l

**Figure 1:**
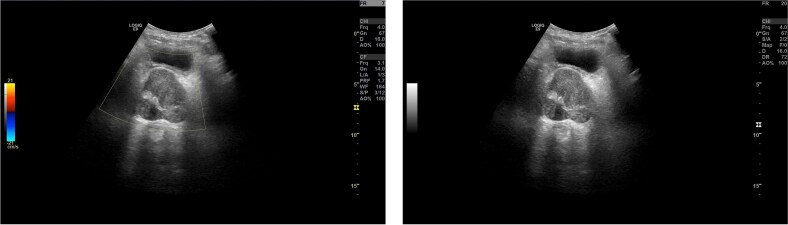
Prostate ultrasound image (transabdominal). The cross-section of the prostate is approximately 4.8 × 5.1 × 4.5 cm in size. The peripheral contour is still clear, and the shape is not regular. Multiple strong echo light spots can be seen inside, and one of them is approximately 0.3 × 0.2 cm in size.

**Figure 2:**
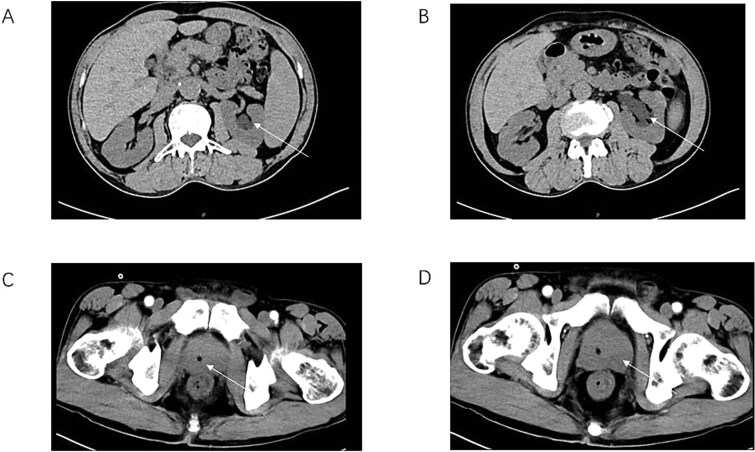
CT of the urinary system (AB) and partial enhancement of the prostate (CD). There was obvious hydronephrosis in the upper renal pelvis of the left ureter in the patient (arrows A&B). The urethra deviates to the right (arrow C), and there is extensive benign prostatic hyperplasia with local mass (arrow D).

**Figure 3:**
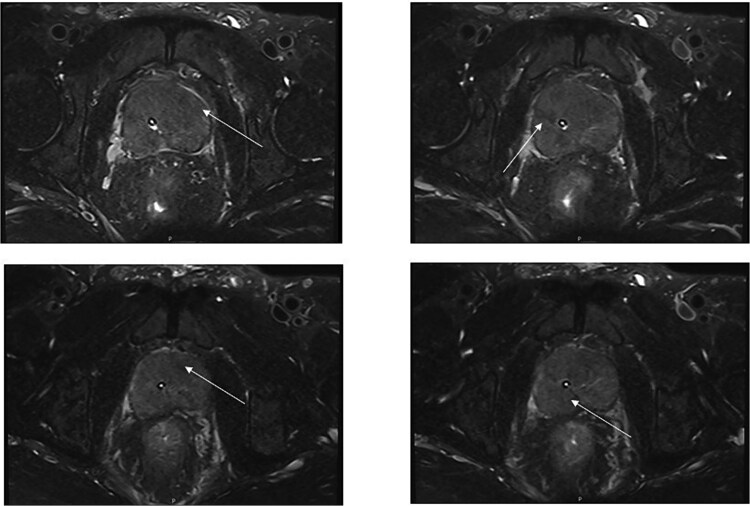
Prostate MRI: The prostate is approximately 53mm × 46mm × 54mm at most; the prostate volume is enlarged and the contour is irregular. The T2WI signals of the bilateral peripheral zones and transition zones of the prostate are significantly decreased and decomposed unclearly, accompanied by the formation of masses (arrows). The urethra deviates to the right; the prostate capsule is intact. The spermatic glands on both sides were not displayed clearly, and the T2WI signal was unevenly decreased. The PI-RADS score is 5. Prostate cancer (mrT3N1Mx) is considered.

**Figure 4:**
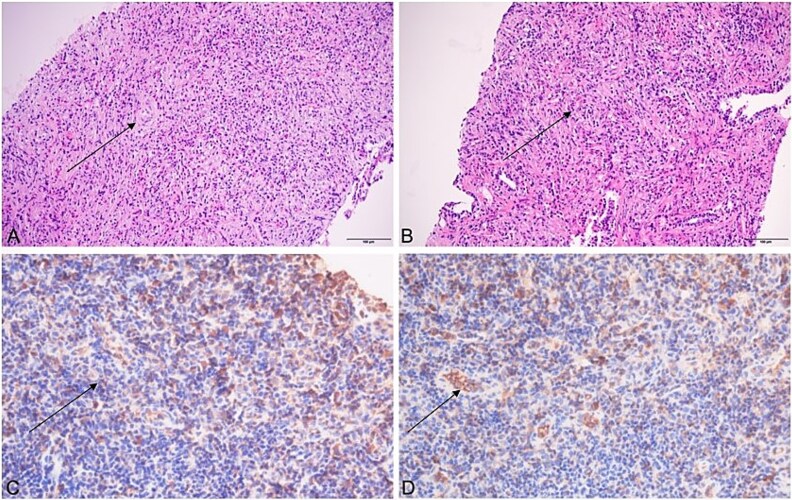
Prostate biopsy tissue (A and B, HE straining 200×). (A) Shows the absence of normal prostatic glands with fibrous hyperplasia in the stroma accompanied by abundant inflammatory cell infiltration, including lymphocytes, plasma cells, and obvious eosinophils. Residual normal prostatic glands, massive lymphocytic infiltration and storiform fibrosis can be seen in the (B). Axillary lymph node biopsy tissue (C and D, immunohistochemistry staining 400×). Immunohistochemistry staining revealing infiltration of IgG(C) and IgG4(D) plasma cells demonstrated.

These findings indicated mild systemic inflammatory activation without evidence of malignant indicators. Imaging Findings: Prostate ultrasound demonstrated prostatic enlargement with hyperechoic nodules (Fig. 1). Subsequent urological computed tomography (CT) revealed hydronephrosis and proximal ureteral dilatation suggestive of neoplastic obstruction, along with prostatic hyperplasia and heterogeneous density nodules (Fig. 2). Given high suspicion for malignancy, pelvic magnetic resonance imaging(MRI) was performed: Prostate dimensions: 53 × 46 × 54 mm; Irregular contour with T2-weighted hypointensity obliterating the peripheral-transitional zone demarcation and mass formation; Prostate Imaging—Reporting and Data System(PI-RADS) 5 classification, radiologically suspicious for prostate cancer (mrT3N1Mx) (Fig. 3) Pathological Evaluation: A 13-core prostate biopsy reveals a significant reduction in the number of prostatic glands, marked interstitial fibrosis with extensive lymphocytic and plasmacytic infiltration. In some areas, storiform or whorled fibrosis and numerous eosinophils are also observed. (Fig. 4A and B). Immunohistochemical analysis demonstrated: Residual acini: PSA(+), P504S(−), CK-H (basal cell+), p63 (basal cell+) Stroma: SMA(+), CD34(−), CK(−), Desmin(−), EMA(−), Ki-67 (labeling index ~ 5%), S-100(−), STAT6(−), ALK(−), CD117(−). These findings conclusively excluded prostatic adenocarcinoma, mesenchymal-derived tumors such as gastrointestinal stromal tumors, solitary fibrous tumors and inflammatory myofibroblastic tumors.

The patient was initially scheduled for partial prostatectomy, with subsequent consideration for radical prostatectomy. However, surgical intervention was suspended due to diagnostic complexities: imaging findings suggestive of malignancy (PI-RADS 5) contradicted the absence of cancer biomarkers (normal PSA, CEA) and cytopathological evidence. Empirical treatment with benign prostatic hyperplasia medications (e.g. tamsulosin) showed no improvement. Concurrent thyroid nodule pathology indicated potential immune-related etiology. Consequently, pure prostatic hyperplasia-related inflammation was deemed insufficient to explain the urinary symptoms. Immunophenotypic analysis of biopsy specimens revealed extensive lymphoproliferative features, prompting comprehensive immunological evaluation.

Serum IgG4 levels were markedly elevated at 25.4 g/l (reference range: 0.03–2.01 g/l). Retrospective immunohistochemical review of prostatic and axillary lymph node biopsies demonstrated (Fig. 4C and D) IgG4/IgG (plasma cells+, IgG4-positive cells > 10/HPF with IgG4:IgG ratio > 40%).

A definitive diagnosis of IgG4-related disease (IgG4-RD) was established. The treatment regimen was determined based on a comprehensive assessment of the patient’s condition and in consultation with the rheumatology department. According to the drug specifications, the recommended dosage of mycophenolate is 0.75–1.0 g twice daily and prednisone 40 mg/day. Initially, we prescribed 1 g twice daily. However, the patient developed significant gastrointestinal symptoms. After discussion with the rheumatology team, the dose was reduced to 0.5 g once daily. With this adjusted dosage, the patient’s condition was effectively controlled.

One-week follow-up demonstrated marked resolution of urinary retention. Two-week post-discharge evaluation revealed: IgG4: 12.6 g/l (50.4% reduction from baseline) Eosinophils: 0.31 × 10^9^/l (86.7% reduction from baseline) This confirms: i) Prostatic IgG4-RD as the underlying etiology. ii)The diagnostic validity of prostate biopsy with IgG4-focused immunohistochemistry. iii)The therapeutic efficacy of glucocorticoid-immunosuppressant combination therapy.

## Discussion

We report a case of IgG4-related disease (IgG4-RD) involving the prostate, characterized by severe urinary retention and hydronephrosis. MRI and CT demonstrated diffuse prostatic enlargement with localized pseudotumoral features. The patient exhibited elevated serum IgG4 levels (25.4 g/l) and histopathological findings of prostatic biopsy revealing dense lymphoplasmacytic infiltration, marked eosinophil infiltration, severe exocrine gland atrophy, and storiform fibrosis with abundant IgG4-positive plasma cells (>10/HPF, IgG4: IgG ratio > 40%), without evidence of malignancy.

IgG4-RD, a recently recognized systemic autoimmune disorder, predominantly involves pancreatic, hepatobiliary, and thyroid tissues. Urological manifestations are uncommon, with renal involvement being relatively more frequent than prostatic involvement. [3] As of May 2025, only 24 English-language cases of IgG4-RD with prostatic involvement have been documented, including 1 case of prostate cancer, 18 cases coexisting with autoimmune pancreatitis (AIP) or IgG4-related cholangitis, 1 case of chronic thyroiditis, and 1 case with regional lymphadenopathy. Our case represents the first reported instance of IgG4-RD directly causing prostatitis through prostatic involvement.

Diagnostic criteria for IgG4-RD require: I) Histopathological evidence of IgG4-positive plasma cell infiltration in affected organs II) Elevated serum IgG4 concentrations III) Favorable response to glucocorticoid therapy IV) Multiorgan involvement [4]. While serum IgG4 remains the most accessible diagnostic marker, elevated levels may occur in diverse conditions, necessitating confirmation through characteristic histopathology. Definitive histological features include dense lymphoplasmacytic infiltrates and IgG4-positive plasma cell enrichment.

In the differential diagnosis of prostatic inflammation, four categories should be considered: I) Acute bacterial prostatitis II) Chronic bacterial prostatitis III) Chronic prostatitis/chronic pelvic pain syndrome (CP/CPPS) IV) Asymptomatic inflammatory prostatitis [5, 6]. Although CP/CPPS accounts for most clinical presentations, this case highlights the importance of distinguishing IgG4-related prostatitis from conventional benign prostatic hyperplasia. Steroid-sensitive IgG4-RD cases may be misdiagnosed as typical chronic prostatitis, leading to unnecessary surgical interventions such as transurethral resection [7]. Retrospective diagnosis often occurs only after multiorgan involvement develops, by which time irreversible procedures may have been performed.

This case underscores the necessity of considering IgG4-RD in patients with prostatic enlargement exhibiting: i) Normal or mildly elevated PSA ii) steroid-responsive symptoms iii) Concurrent immune dysregulation.

The diagnostic paradigm of prostate biopsy with IgG4-specific immunohistochemistry provides a non-surgical approach to avoid iatrogenic harm, offering new perspectives for managing atypical prostatic hyperplasia.
